# Metastatic Gastrointestinal Adenocarcinoma with Osteoblastic Activity: A Case Report of Esophageal and Colonic Primaries

**DOI:** 10.1155/2016/1934759

**Published:** 2016-09-22

**Authors:** Brett Matthew Lowenthal, Ahmed S. Shabaik, Mark A. Valasek

**Affiliations:** Department of Pathology, University of California, San Diego, 200 West Arbor Drive, MC 8320, San Diego, CA 92103, USA

## Abstract

Adenocarcinoma with osteoblastic metastases is classically seen in prostate, breast, and lung primaries. Less common primary sites include thyroid, kidney, and stomach. We present two cases of primary gastrointestinal adenocarcinoma with metastatic osteoblastic activity from two previously unreported sites. The first case represents an esophageal adenocarcinoma arising in a background of intestinal metaplasia that metastasized with osteoblastic activity to the deltoid muscle. The second case demonstrates a Stage IV sigmoid colon adenocarcinoma with osteoblastic metastases to the liver and lymph nodes. The findings indicate that metastases from various gastrointestinal primary adenocarcinomas can have prominent bone formation.

## 1. Introduction

Metastatic adenocarcinoma with osteoblastic activity is classically seen with breast, prostate, and lung carcinomas and less commonly with renal, thyroid, and gastric carcinomas [1]. Despite gastric carcinomas being the most common gastrointestinal malignancy to metastasize with osteoblastic activity, primary gastric carcinoma is a very rare occurrence [1, 2]. Metastatic adenocarcinoma of esophageal or colonic primary has not been described in the literature to produce extraskeletal metastases with osteoblastic bone formation. Here we present two cases of metastatic gastrointestinal adenocarcinoma, one esophageal primary (Case 1) and one colonic primary (Case 2), to extraskeletal sites with osteoblastic bone formation and ossification closely associated with the neoplastic glands.

## 2. Case Presentation

### 2.1. Case 1

**Figure 1:**
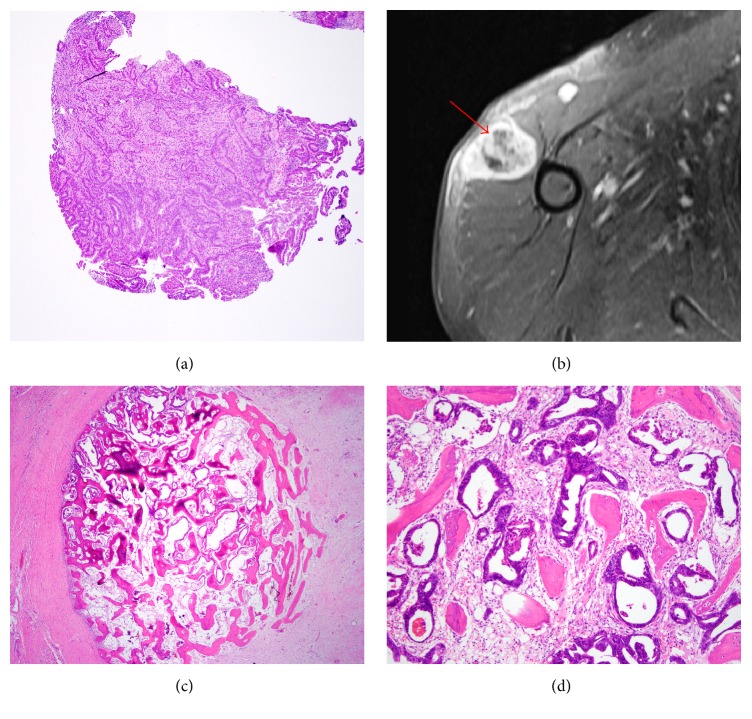
Case 1 histologic features of the esophageal mass and soft tissue mass. (a) Biopsy of the distal esophageal mass demonstrated infiltrating moderately differentiated neoplastic glands consistent with primary esophageal adenocarcinoma in a background of goblet cell (intestinal) metaplasia (H&E, 40x). (b) MRI of the deltoid muscle soft tissue mass highlighted bone and calcifications within the lesion that is distinctly separate from the humerus (red arrow). (c) Resection of the soft tissue mass at low power magnification showed a well-circumscribed mass of neoplastic glands closely associated with osteoblastic activity within skeletal muscle and fibrosis (H&E, 20x). (d) Resection of soft tissue mass at higher magnification highlighted moderately differentiated neoplastic glands with osteoblastic activity, but lacking bone marrow elements (H&E, 100x).

A 49-year-old male with a multiple year history of gastroesophageal reflux disease presented with a six-month history of a 50-pound unintentional weight loss with progressive dyspepsia, reflux, and dysphagia of solids and some liquids. Esophagogastroduodenoscopy identified a large obstructing esophageal mass located at 35–40 cm with unremarkable stomach and duodenum. The distal esophageal biopsy showed invasive moderately differentiated esophageal adenocarcinoma in the setting of Barrett esophagus (Figure 1(a)). An esophageal stent was placed to help alleviate the patient's symptomatology.

**Figure 2:**
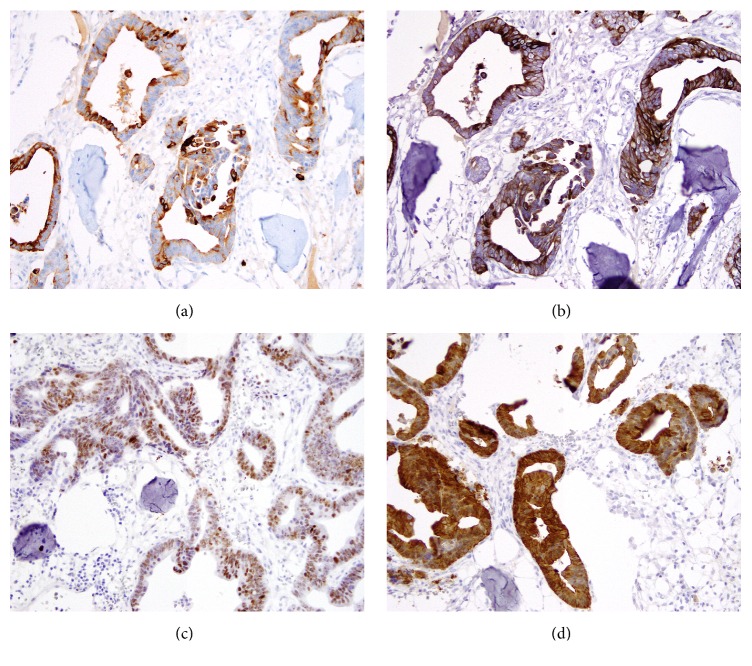
Case 1 immunohistochemical features of the soft tissue mass confirming metastatic focus of esophageal adenocarcinoma. The neoplastic glandular cells were diffusely and strongly positive for CK7 ((a) 200x), CK20 ((b) 200x), CDX-2 ((c) 200x), and Villin ((d) 200x).

During the initial exam, the patient reported a firm mass overlying his right shoulder. Magnetic Resonance Imaging revealed a 4.7 cm heterogeneous soft tissue mass within the right deltoid muscle lateral to and separate from the humeral shaft (Figure 1(b)). The radiologic differential diagnosis included sarcoma, vascular origin tumor, and less likely metastasis. The patient underwent an extensive metastatic workup to reveal no additional metastases at that time. Soft tissue resection showed a superficial calcified mass within the subcutaneous and muscular tissue. Histologic examination of this soft tissue mass demonstrated neoplastic glandular cells intimately associated with osteoblastic bone formation with no bone marrow elements (Figures 1(c) and 1(d)). Immunohistochemical studies demonstrated that these neoplastic glandular cells were diffusely positive for CK7, CK20, CDX-2, and Villin (Figures 2(a)–2(d)) (Ventana Medical Systems, Tucson, AZ). When compared to the initial distal esophageal biopsy, the soft tissue mass morphology and immunohistochemical profile were consistent with metastatic esophageal adenocarcinoma.

Since the patient's initial presentation of dysphagia and soft tissue mass, the patient has undergone multiple rounds of chemotherapy and radiation. He since has developed progressive disease with new metastases to the pancreas, iliacus muscle, and axillary lymph nodes.

### 2.2. Case 2

**Figure 3:**
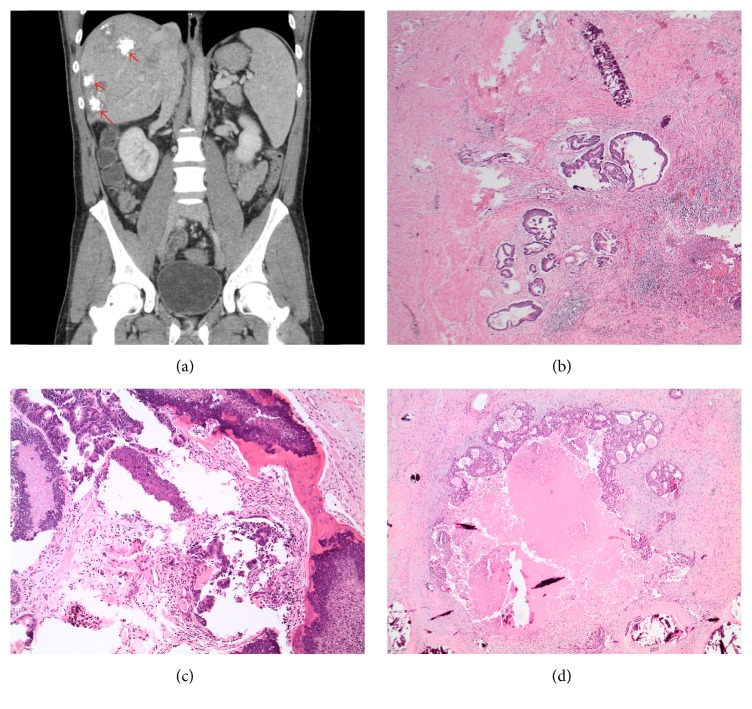
Case 2 histologic features of the sigmoid colon mass, lymph node, and liver masses. (a) CT scan of the abdomen demonstrated liver metastases with marked calcifications within the lesion (red arrow). (b) Left hemicolectomy of the sigmoid colon mass demonstrated moderately differentiated infiltrating neoplastic glands within ulcerated colonic mucosa consistent with primary colonic adenocarcinoma with associated calcifications (H&E, 40x). (c) Resection of lymph node showed foci of metastatic neoplastic glandular cells of colonic primary with associated osteoblastic activity demonstrated by bone formation and calcifications (H&E, 100x). (d) Resections of the liver masses highlighted metastatic neoplastic glands of colonic primary with associated osteoblastic activity demonstrated by calcifications (H&E, 40x).

A 38-year-old previously healthy male presented to an outside hospital with bright red blood per rectum with associated fatigue and anemia. He underwent esophagogastroduodenoscopy and colonoscopy. The colonoscopy showed a sigmoid mass with histology confirming adenocarcinoma. The upper endoscopy was unremarkable. Staging computed tomography scans of the chest, abdomen, and pelvis demonstrated diffuse metastatic disease in the liver, colonic lymph nodes, and cardiophrenic lymph nodes. The liver masses showed significant calcifications on the imaging (Figure 3(a)).

At this point, he was transferred to our hospital for further management and initiation of preoperative chemotherapy. He demonstrated radiologic response with a decrease in size of multiple liver lesions. A left hemicolectomy, cholecystectomy, and liver excisions were performed. Gross pathologic examination of the hemicolectomy revealed a 2.3 cm circumferential ulcerated mass at the sigmoid colon that was firm and very difficult to section. Histology confirmed the previously reported invasive colonic adenocarcinoma with metastases to lymph nodes and liver. The primary colonic site (Figure 3(b)), metastatic lymph nodes (Figure 3(c)), and liver (Figure 3(d)) metastases demonstrated moderately differentiated neoplastic glandular cells with associated osteoblastic activity, ossification, calcification, and fibrosis.

Staging workup revealed Stage IV colon cancer. The patient tolerated the operation and is now postoperative three months with an uncomplicated course. He is doing well and currently on a postoperative chemotherapy regimen.

## 3. Discussion

Adenocarcinoma of gastrointestinal origin commonly metastasizes to the lymph nodes, liver, and numerous additional sites. The only gastrointestinal carcinomas documented to develop metastases with osteoblastic activity are from the stomach and occur very rarely. Breast and prostate carcinomas are typically seen to develop metastases that are osteoblastic in nature [2, 3]. Other much less common sites of origin to develop osteoblastic metastases include lung, kidney, and thyroid carcinomas [2–5]. One paper reported pancreas as an originating site for osteoblastic metastases [6]. Gastric adenocarcinoma with osteoblastic metastases has only been reported in a handful of case reports [2–5].

After an extensive review of the literature of adenocarcinomas producing disseminated extraskeletal osteoblastic metastases, we have not found any cases of carcinomas demonstrating this feature and originating from the esophagus or colon. Primary esophageal and colonic adenocarcinoma has been reported to metastasize to osseous sites with osteoblastic activity [7, 8]. Both of our patients, one with esophageal adenocarcinoma and the other with colonic adenocarcinoma, demonstrate this rare and bizarre phenotype of osteoblastic metastases. The first patient presented with a large nearly obstructing carcinoma located in the esophagus with unremarkable stomach and duodenum on endoscopy. During the history and physical examination, it was discovered that he additionally had a firm soft tissue mass initially thought to be a second primary tumor (i.e., sarcoma or vascular origin tumor) after clinical and radiological evaluation. Upon resection, it was found to be an extensively calcified mass demonstrating an osteoblastic metastasis of the esophageal adenocarcinoma primary. In the second case, the patient initially presented at a young age with hematochezia. Upper and lower endoscopy revealed a large circumferential ulcerated sigmoid colon mass. Additional workup revealed metastatic disease to lymph nodes and multiple liver sites. The surgery confirmed the diagnostic osteoblastic metastases to both the lymph nodes and liver.

It is interesting to note that special AT-rich sequence-binding protein 2 (SATB2), which is a nuclear matrix protein that regulates osteoblastic differentiation [9, 10], can be expressed in gastrointestinal adenocarcinomas. It has been shown that SATB2 germline deletion mutation in both mice and humans leads to dysregulation of osteoblasts [11–13]. Although SATB2 is a sensitive marker of osteoblastic osteosarcomas, it is not a specific marker and can be seen in adenocarcinomas, especially of colonic origin [14]. In one study, SATB2 expression was diffuse and strongly positive in 96.8% of adenocarcinomas of colorectal origin and positive in only 6.7%, 0%, and 4.2% of adenocarcinomas of esophagus, stomach, and pancreas, respectively [15].

In summary, we presented two cases of metastatic extraskeletal adenocarcinoma with osteoblastic activity. Although commonly seen in breast and prostate and rarely seen in kidney, thyroid, and stomach, our cases are the first to be reported originating from the esophagus and the colon.
